# Kinetic properties and stability of glucose dehydrogenase from *Bacillus amyloliquefaciens* SB5 and its potential for cofactor regeneration

**DOI:** 10.1186/s13568-015-0157-9

**Published:** 2015-11-04

**Authors:** Thunyarat Pongtharangkul, Pattra Chuekitkumchorn, Nhuengtida Suwanampa, Panwajee Payongsri, Kohsuke Honda, Watanalai Panbangred

**Affiliations:** Department of Biotechnology, Faculty of Science, Mahidol University, Bangkok, 10400 Thailand; Department of Biotechnology, Graduate School of Engineering, Osaka University, Osaka, Japan

**Keywords:** *gdh*, Glucose 1-dehydrogenase, Cofactor regeneration, *Bacillus amyloliquefaciens*

## Abstract

Glucose dehydrogenases (GluDH) from *Bacillus* species offer several advantages over other NAD(P)H regeneration systems including high stability, inexpensive substrate, thermodynamically favorable reaction and flexibility to regenerate both NADH and NADPH. In this research, characteristics of GluDH from *Bacillus amyloliquefaciens* SB5 (GluDH-BA) was reported for the first time. Despite a highly similar amino acid sequence when comparing with GluDH from *Bacillus subtilis* (GluDH-BS), GluDH-BA exhibited significantly higher specific activity (4.7-fold) and stability when pH was higher than 6. While an optimum activity of GluDH-BA was observed at a temperature of 50 °C, the enzyme was stable only up to 42 °C. GluDH-BA exhibited an extreme tolerance towards *n*-hexane and its respective alcohols. The productivity of GluDH obtained in this study (8.42 mg-GluDH/g-wet cells; 1035 U/g-wet cells) was among the highest productivity reported for recombinant *E. coli*. With its low *K*_M_-value towards glucose (5.5 mM) and NADP^+^ (0.05 mM), GluDH-BA was highly suitable for in vivo applications. In this work, a recombinant solvent-tolerant *B. subtilis* BA overexpressing GluDH-BA was developed and evaluated by coupling with *B. subtilis* overexpressing an enzyme P450 BM3 F87V for a whole-cell hydroxylation of *n*-hexane. Significantly higher products obtained clearly proved that *B. subtilis* BA was an effective cofactor regenerator, a valuable asset for bioproduction of value-added chemicals.

## Introduction

Bio-based chemical production has been depicted as a promising approach to a sustainable chemical manufacturing. Biocatalytic oxidoreductions hold great potential for industrial production of enentiopure chemicals and pharmaceuticals. Most oxidoreductase enzymes, however, require prohibitively expensive nicotinamide cofactors (NADH or NADPH). To date, the use of NADPH-dependent catalysts has been severely limited by an absence of efficient methods to recycling the cofactor. An efficient cofactor regeneration system is, therefore, one of the most critical requirements prior to a commercialization of biocatalytic oxidoreductions. Thus far, biological approaches using either isolated enzyme or whole-cell biocatalyst seem to exhibit the highest potential for commercial applications (de Wildeman et al. 2007).

Glucose dehydrogenase (GluDH; EC 1.1.1.47) catalyzes the oxidation of β-d-glucose to β-D-glucono-1,5-lactone with simultaneous reduction of the cofactor NAD(P)^+^ to NAD(P)H. The enzyme occurs in a variety of organisms such as *Bacillus megaterium*, *Bacillus subtilis*, *Gluconobacter suboxydans*, *Halobacterium mediterranei*, *Thermoplasma acidophilum*, and *Sulfolobus solfataricus*. GluDHs from different organisms show diverse biochemical properties (e.g. activity and stability) and preference towards the cofactors (NAD^+^ and NADP^+^). Amino acid sequence alignment indicated that NAD(P)^+^-dependent GluDHs from the *Bacillus* species, belonging to the extended superfamily of short-chain dehydrogenases/reductases (SDR) (Nishiya et al. 2004), have more than 80 % homology (Xu et al. 2007). GluDH from *B. subtilis* (GluDH-BS) has been used extensively for in vivo NAD(P)H regeneration (Zhu et al. 2006; Schewe et al. 2008; Zhang et al. 2009, 2011; Richter et al. 2010), mainly because of its dual cofactor specificity and ease of expression in a commonly used *Escherichia coli* hosts.

Despite its successful application in a bioproduction of epoxyhexane (Siriphongphaew et al. 2012), in this study, GluDH from *Bacillus amyloliquefaciens* SB5 (GluDH-BA) was studied in detail in order to evaluate its full potential in biocatalysis. A GluDH-encoding gene from *B. amyloliquefaciens* SB5 has been cloned and expressed in both *E. coli* and *B. subtilis*. The kinetics and biochemical properties of the purified enzyme were evaluated. To our knowledge, this is the first report on characteristics of GluDH from *B. amyloliquefaciens*. Moreover, for an in vivo application, a recombinant *B. subtilis* 168 overexpressing GluDH-BA (referred as *B. subtilis* BA) was evaluated as a whole-cell cofactor regenerating biocatalyst for hydroxylation of *n*-hexane by coupling with *B. subtilis* overexpressing P450 BM3 F87V.

## Methods

### Chemicals and media

All chemicals used were of analytical grade and commercially available from Sigma-Aldrich (USA). Luria–Bertani (LB) and Tryptic soy broth (TSB) were used for cultivation and storage of working culture. For solid media, 1.5 % Bacto Agar (Lab M, Lancashire, UK) was added. Ampicillin (Amp) at 50 μg/μl and tetracycline (Tet) at 20 μg/μl were used for selection and cultivation of recombinant *E. coli* and *B. subtilis*, respectively.

### Strains, plasmids and culture conditions

*B. amyloliquefaciens* SB5 was previously isolated from a petroleum contaminated soil using an enrichment technique with benzene as a screening agent. Its identity was confirmed using bioMerieux api™ 20E and 50CHB/E test strips (bioMerieux, Marcy L’Etoile, France) and 16s rDNA sequencing. The strain was deposited to TISTR Microbiological Resources Centre (Pathumthani, Thailand) under the accession number TISTR2086. *Bacillus subtilis* 168 (BGSC 1A1) was kindly provided by Bacillus Genetic Stock Center (BGSC, Columbus, OH). Recombinant *B. subtilis* 3C5N (pHBGA), referred as *B. subtilis* B-7, was developed previously by Siriphongphaew et al. (2012).

### Construction of recombinant plasmids for an expression of glucose 1-dehydrogenase in *E. coli*

Standard procedures according to Sambrook and Russell (2001) were used for DNA manipulation of *E. coli*. Full length *gdh* genes were amplified by high-fidelity PCR from genomic DNA of *B. subtilis* 168 and *B. amyloliquefaciens* SB5 using primers designed based on known sequences of *gdh* from *B. subtilis* 168 (Accession No. NC000964) and *B. amyloliquefacien* FZB42 (Accession No. ABS72817) (Table 1; primers no. 1, 2 and 3). To facilitate the PCR amplification, the genomic DNA was digested with *Hind*III before being used as a template. The amplified fragments (0.8 kb) were retrieved after running on a 1.5 % agarose gel and then cloned into pJET1.2/blunt-end cloning vector (Thermo Scientific, IL, USA), resulting in plasmids named as pJET-*gdh-bs* and pJET-*gdh-ba*. The nucleotide sequence of *gdh* from *B. amyloliquefaciens* SB5 (*gdh*-*ba*) was determined and compared with those of other bacteria. In order to simplify the purification process, the *gdh*-*ba* fragment was then amplified with primers in which nucleotides coding for a 6× His-tag were placed immediately after the start codon (Table 1; primers no. 4 and 5). The fragment was double digested with *Nde*I and *Xho*I and ligated into pET23b + (Novagen) digested with the same pair of enzymes. The plasmid pET23b-*gdh-ba* was introduced into *E. coli* DH5α and the sequence of *gdh* genes were confirmed by sequencing before subcloning into *E. coli* BL21(DE3).

**Table 1:** Primers used in this study

No.	Primer	Sequence
1	*gdh-bs*-F-BamHI	5′CCGGATCCATGTATCCGGATTTAAAAGGAA3′
2	*gdh-ba*-F-BamHI	5′CCGGATCCATGTACACGGATTTAAAAGGAA3′
3	*gdh*-R-KpnI	5′CTGGTACCTTATCCGCGGCCTG3′
4	*gdh-ba*-F-NdeI-His	5′CGCCATATGCACCACCACCACCACCACTACACGGATTTAAAAGGAAAAG3′
5	*gdh*-R-XhoI	5′CCGCTCGAGTTATCCGCGGCCTGCCTGGAATG3′
6	*gdh-bs*-F-BsrGI	5′CCTGTACAATGTATCCGGATTTAAAAGGAA3′
7	*gdh-ba*-F-BsrGI	5′CCTGTACAATGTACACGGATTTAAAAGGAA3′

Restriction sites are underlined

### Purification of GluDH-BA

The entire purification process was performed at 4 °C. *Escherichia coli* BL21(DE3) harboring the plasmid pET23b-*gdh-ba* was cultivated in an auto-induction medium (containing 0.5 g/L glucose, 6 g/L glycerol, 2 g/L lactose, 10 g/L peptone, 5 g/L yeast extract, 5 g/L sodium chloride, 6 g/L Na_2_HPO_4_ and 3 g/L KH_2_PO_4_) at 37 °C, 200 rpm for 6 h and then at 20 °C, 200 rpm for 14 h. Cells were harvested, resuspended in phosphate buffer (pH 8) and broken with Bead beater-1 (0.25 g of 1-mm ϕ glass bead per ml; 90 s per cycle at 4300 rpm; 5 cycles; chilled on ice for 1 min between each cycle) (Biospec, OK, USA). After centrifugation at 11,337×*g*, 4 °C for 30 min, the supernatant was collected and purified using PrepEase^®^ Histidine-Tagged Protein Purification Kits – High Specificity according to the recommended protocol (usb, OH, USA). The purified enzyme was diluted (5×) with Citrate-Phosphate-Borate or CPB buffer (pH 6) and then concentrated using a spin filter with MWCO of 10 kDa prior to a storage at −80 °C. The protein concentration was determined by the Bradford assay (Bradford 1976) using bovine serum albumin as a standard. SDS-PAGE was carried out on a 4–12 % Bis–Tris Gel (NuPAGE Novex, Thermo Scientific, IL, USA) at a constant current (15 mA/gel). PageRuler™ Prestained Protein Ladder (Thermo Scientific, IL, USA) was used as a molecular mass marker. After electrophoresis, the gel was stained with Coomassie brilliant blue R-250 (Laemmli 1970) and de-stained by soaking in the methanol:acetic acid solution.

### Construction of recombinant plasmids and expression of glucose 1-dehydrogenase in *B. subtilis*

*Bacillus subtilis* 168, previously reported to exhibit an organic solvent-tolerant property (Siriphongphaew et al. 2012), was selected as a host for development of a whole-cell cofactor regenerator. The *gdh* fragments were amplified from the plasmid pJET-*gdh-bs* and pJET-*gdh-ba* using primers containing restriction sites for *BsrG*I and *Kpn*I (Table 1; primers no. 3, 6 and 7). After a double digestion with *BsrG*I and *Kpn*I, the fragments were inserted into a *Bacillus* expression vector pHP2N. Plasmid pHP2N was constructed based on a plasmid backbone of *E. coli*-*Bacillus* shuttle vector pHY300PLK (TaKaRa, Shiga, Japan) with an insertion of a strong P2 promoter from a commercial expression plasmid pNCMO2 (TaKaRa, Shiga, Japan). The resulting plasmids, named as pHGBS and pHGBA, were introduced into *B. subtilis* 168 by electroporation using a protocol described previously (Siriphongphaew et al. 2012). Successful transformation was checked by a colony PCR as well as a restriction analysis. The recombinant *B. subtilis* 168 overexpressing GluDH-BS and GluDH-BA were referred as *B. subtilis* BS and *B. subtilis* BA, respectively.

### Crude enzyme preparation

Crude enzyme extract was prepared from *B. subtilis* BS and *B. subtilis* BA as follow. Cells were cultivated in LB_tet_ at 37 °C, 200 rpm for 16 h, harvested by centrifugation at 11,337×*g* for 10 min, washed twice and re-suspended in CPB buffer to an optical density (600 nm) of 10. Cells were broken with Bead beater as previously described and supernatant was collected after centrifugation at 11,337×*g* for 10 min.

### GluDH activity assay

GluDH activity was assayed by measuring the absorbance of NAD(P)H at 340 nm. Unless specified otherwise, the reaction was performed in 50 mM Tris–Cl buffer (pH 8) with 0.5 mM NAD(P)^+^ and 50 mM glucose at 37 °C. The concentration (U/ml) was calculated using the millimolar extinction coefficient (*ε*_340_) of 6.22. One unit (U) of GluDH activity was defined as the formation of 1 μmole of NAD(P)H per minute and the specific activity was reported in terms of U/g-CDW as well as U/mg-protein.

### Effect of pH and temperature on activity and stability of purified GluDH-BA and intracellular GluDH-BA

Effect of pH on specific activity of purified GluDH-BA was determined by measuring the activity at 37 °C using CPB buffer with a pH range from 5 to 10. The effect of pH on enzyme stability was determined from the residual activity (%) after incubating the purified enzyme in the CPB buffer with a specified pH at 30 °C for 6 h. The stability of the enzyme inside the whole-cell biocatalyst was determined in a similar manner except that the cell suspension (OD_600_ = 10) was incubated in the specified buffer for 6 h before being used for preparation of crude extract. For comparison, the stability of intracellular GluDH-BA was compared with those of crude GluDH-BS and crude GluDH-BA prepared from *B. subtilis* BS and *B. subtilis* BA, respectively.

Effect of temperature on activity and stability of the purified GluDH-BA was evaluated using CPB (pH 6) over the temperature range of 10–60 °C. The effect of temperature on stability was determined from the residual activity after incubating the enzyme at a specified temperature for 6 h.

### Effect of organic solvents on stability of GluDH-BS and GluDH-BA

Effect of various organic solvents (DMSO, acetone, ethanol, *n*-butanol, *n*-hexane, 1-hexanol and 2-hexanol) on stability of the purified GluDH-BS and GluDH-BA were evaluated by incubating the enzyme with either 10 or 50 % (v/v) of the specified organic solvent at 30 °C for 1 h. The GluDH activity was then assayed using CPB (pH 6) at 37 °C. Relative activity (%) was calculated based on a control in which water was added instead of the organic solvent.

### Steady-state kinetics

Apparent *K*_M_ and *V*_max_ value of the purified GluDH-BA were determined using various substrate concentrations (10-250 mM of glucose) at a fixed concentration of the acceptor (0.5 mM of NAD^+^ or NADP^+^). Double reciprocal plot was used to determine *K*_M_ and *V*_max_ and only the concentrations that did not cause substrate inhibition were used. The initial rate was determined by linear regression as a slope of the linear plot between NAD(P)H formation and time.

### Application of *B. subtilis* BA as a whole-cell cofactor regenerator

**Fig. 1:**
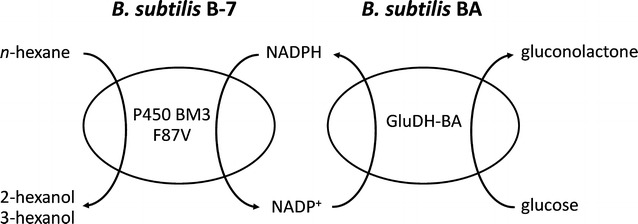
Hydroxylation of *n*-hexane using a coupling whole-cell system consisting of *B. subtilis* B-7 and *B. subtilis* BA

*Bacillus subtilis* BA was evaluated as a whole-cell cofactor regenerating biocatalyst for hydroxylation of *n*-hexane by coupling with *B. subtilis* 3C5N overexpressing P450 BM3 F87V (referred as *B. subtilis* B-7) (Fig. 1). *Bacillus subtilis* B-7 and *B. subtilis* BA were cultivated in LB_tet_ at 37 °C, 200 rpm for 12 h, harvested, washed, and resuspended in a reaction buffer (100 mM KPi buffer, pH 7 supplemented with 110 mM glucose) to OD_600_ of 25. For permeabilized *B. subtilis* B-7, 1 ml of the cell suspension was treated with 10 μl of toluene for 1 h before being used for a bioconversion in which 0.2 mM NADP^+^ was supplemented. Then, 100 mM of *n*-hexane was added into the cell suspension (1 ml) to start the reaction and the mixture was incubated at 30 °C, 200 rpm for 3 h. Finally, hexanols (2- and 3-hexanol) were extracted using 400 µL of butyl acetate and quantified by using GC (GC-2014, Shimadzu, Japan) equipped with a flame ionization detector (FID) and a DB-WAX column (30 m in length, 0.25 mm inner diameter, and 0.25 µm film thickness; J & W Scientific, Folsom, CA, USA). Helium was used as a carrier gas at a total flow rate of 1 ml/min. The sample was injected using a split mode with a split ratio of 5:1. The injector and FID detector temperature were set at 250 and 260 °C, respectively. The column temperature profile was 40–160 °C at 8 °C/min, 160–240 °C at 20 °C/min and 240 °C for 5 min. The calibration curves were constructed based on the peak areas of commercial standards prepared in Kpi buffer (pH 7) and extracted using the same procedure as samples.

### Statistical analysis

All experiments were performed in duplicate. Statistical analysis including ANOVA and multiple comparisons (Tukey’s test) were performed using Minitab (Release 15, State College, PA, USA).

### Strain and amino acid sequence

*B. amyloliquefaciens* SB5 has been deposited to Thailand Institute of Scientific and Technological Research or TISTR (Pathumthani, Thailand) with the accession number TISTR2086. An amino acid sequence of its *gdh* gene has been submitted to the GenBank nucleotide sequence database (NCBI) under an accession number JQ305165.

## Results

### Amino acid sequence and structural analysis of GluDH from *B. amyloliquefaciens* SB5

Interestingly, *B. amyloliquefaciens* SB5 exhibited a highly active NAD(P)H-regenerating activity when compared with the most studied *Bacillus* host *B. subtilis* 168 and *B. subtilis* 3C5N, our organic solvent-tolerant host previously reported (Siriphongphaew et al. 2012) (Table 2). A full-length *gdh* gene (786 bp) of *B. amyloliquefaciens* SB5 was successfully amplified from its genomic DNA using primers designed based on a *gdh* gene of a closely related strain, *B. amyloliquefaciens* FZB42 (GenBank accession number ABS72817.1).

**Table 2:** GluDH activity of cell-free extracts prepared from wild-type strains and their recombinants

Microorganism	GluDH activity^a^	
	U/g-CDW	U/mg-total protein
*B. subtilis* 168	0.99 ± 0.44	0.008 ± 0.001
*B. subtilis* 3C5N	0.22 ± 0.05	0.002 ± 0.000
*B. amyloliquefaciens* SB5	15.8 ± 1.90	0.166 ± 0.085
*B. subtilis* B-7^b^	0.02 ± 0.02	0.0002 ± 0.0002
*B. subtilis* BS^c^	6.84 ± 0.59	0.020 ± 0.003
*B. subtilis* BA^d^	1601 ± 146	6.24 ± 0.39

^a^The activity was determined using Tris–Cl (pH 8) at 37 °C

^b^ *B. subtilis* B-7 was a recombinant *B. subtilis* 3C5N overexpressing P450 BM3 F87V (Siriphongphaew et al. 2012)

^c^ *B. subtilis* BS was a recombinant *B. subtilis* 168 overexpressing GluDH-BS from itself

^d^ *B. subtilis* BA was a recombinant *B. subtilis* 168 overexpressing GluDH-BA from *B. amyloliquefaciens* SB5

**Fig. 2:**
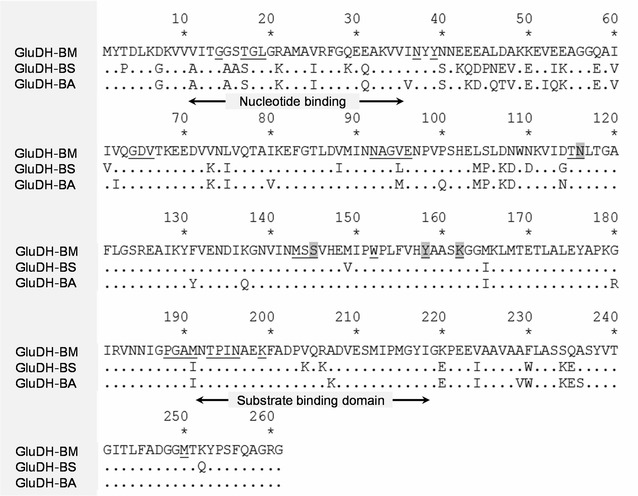
Amino acid sequence alignment of GluDH-BA (JQ305165) from *B. amyloliquefaciens* SB5 with GluDH-BM (P39485) from *B. megaterium* and GluDH-BS (P12310) from *B. subtilis* 168. Amino acids located 4 Å away from glucose and NAD^+^ were defined as an active site channel and presented as *underlined alphabets*. Nucleotide binding site (amino acid 11–35), the catalytic tetrad (amino acid 116, 145, 158, and 162; highlighted in *grey*) and the amino acids involving in substrate binding (amino acid 192–218) are indicated in the figure

Alignment of the deduced amino acid sequence of GluDH-BA (261 aa) with its bacterial counterparts showed highest similarity with GluDH from *B. amyloliquefaciens* FZB42 (99 %) and high similarity of 89 % with GluDH from *B. subtilis* 168 (UniProtKB entry number P12310). The catalytic tetrad, Asn116-Ser145-Tyr158-Lys162, conserved among other GluDHs was also present (Fig. 2). Both GluDHs, GluDH-BS from *B. subtilis* 168 and GluDH-BA from *B. amyloliquefaciens* SB5, showed high level of similarity (82 and 83 %, respectively) toward the GluDH IV (P39485), a GluDH with a known crystal structure (Nishioka et al. 2012). With this high level of sequence similarity, proteins would have the same folding, architecture and overall quaternary structure even if GluDH-BS and GluDH-BA exhibited different kinetic properties as illustrated in Table 3.

**Table 3:** Kinetic constants of purified GluDH from *B. amyloliquefaciens* SB5 comparing to those reported in the literature

Microorganism	*K*_M_ value (mM)			Turnover number (1/s)^a^	Specific activity (U/mg-protein)	Optimal pH	References
	NAD^+^	NADP^+^	β-D-glucose^a^				
*Bacillus subtilis*	0.23	0.26	42.9	n.a.^c^	26	9	Fujita et al. (1977)
*Gluconobacter suboxydans*	n.d.^b^	0.01	5	n.a.^c^	140	6	Adachi et al. (1980)
*Sulfolobus solfataricus*	1.2	0.03	0.44	48	437	8	Giardina et al. (1986)
*Bacillus megaterium*	0.37	0.03	11	260	n.a.^c^	6.5	Yamamoto et al. (1990)
*Lysinibacillus sphaericus* G10	0.09	0.07	5.1	93	205	9.5	Ding et al. (2011)
*Bacillus amyloliquefaciens* SB5	0.25 ± 0.03	0.05 ± 0.004	5.5 ± 0.28	70 ± 0.3	123 ± 0.5	10	This study

All kinetic parameters of GluDH-BA are presented as Avg ± SD

^a^With NADP^+^

^b^No activity with NAD^+^

^c^Data not available

**Fig. 3:**
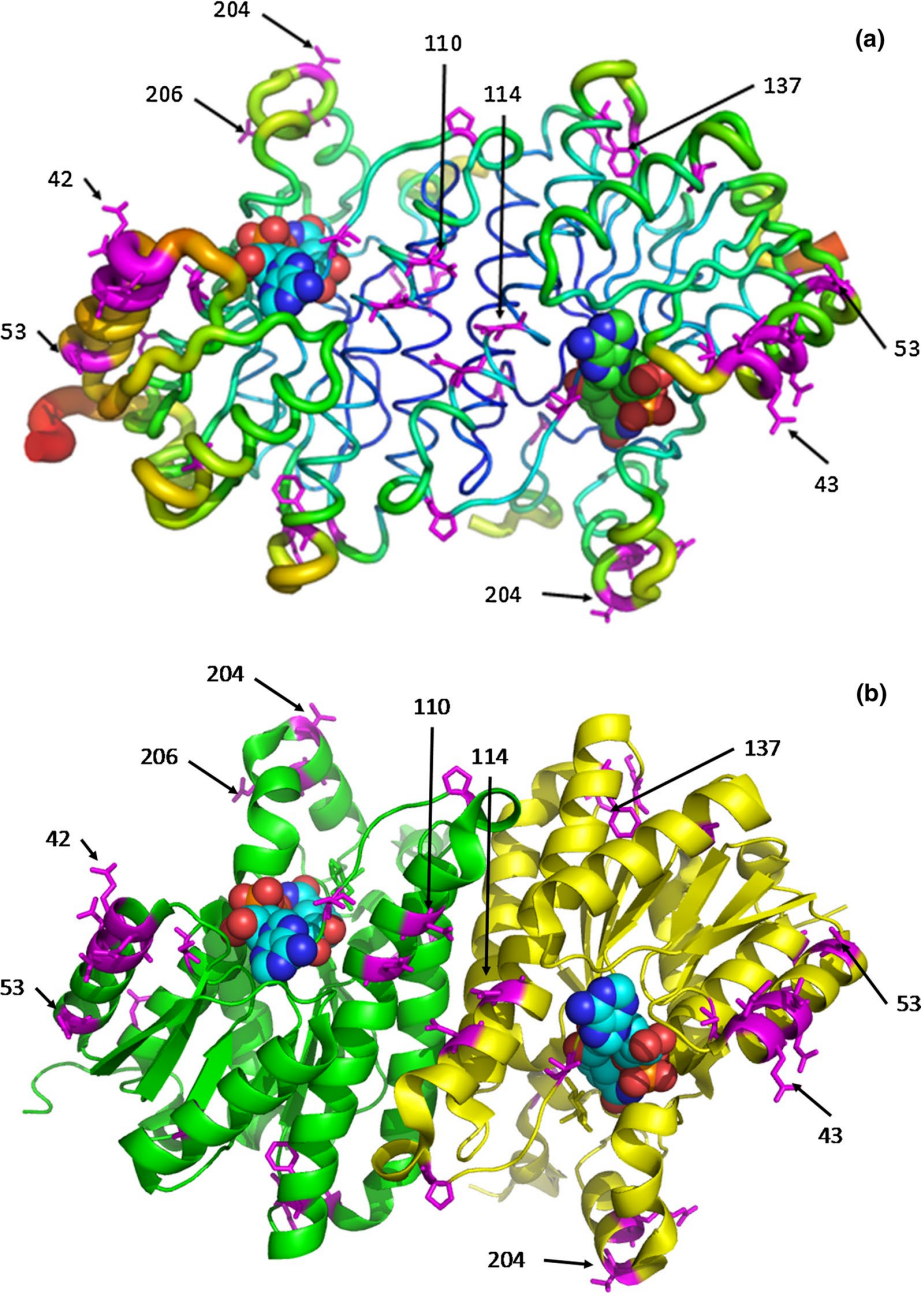
Locations of amino acid variations between GluDH-BS and GluDH-BA (shown as *magenta stick*) in the crystal structure of GluDH from *B. megaterium* (PDB ID: 3AY6) (Nishioka et al., 2012). NAD^+^ is shown as *sphere*. Each subunit was highlighted as *green* and *yellow*

All GluDHs compared in Fig. 2 belong to the extended superfamily of short-chain dehydrogenases/reductases (SDR) with an expected size of approximately 28 kDa. The crystal structure of GluDH IV (referred as GluDH-BM in this study) from *Bacillus megaterium* (PDB ID 3AY6) was illustrated in Fig. 3. Crystal structure analysis allowed us to identify amino acid residues that constitute the active site channel, defined as any amino acids located 4 Å from glucose and NAD^+^. Twenty-nine amino acids that are different between GluDH-BA and GluDH-BS are shown as magenta stick (Fig. 3). Together with sequence alignment in Fig. 2, it was clear that majority of the differences between these two GluDHs were outside of the active site channel except only an amino acid at position 95. The fact that the most variable region was at the nucleotide binding domain may contributes to an extremely low *K*_M_ towards NADP^+^ observed in GluDH-BA.

From structural analysis, most of the variable amino acids are located on rather highly flexible regions (Fig. 3b). Interestingly, there were cases where a mutation at the flexible regions improved the thermostability of an enzyme (Reetz et al. 2006). In order to evaluate the effect of amino acid variation between GluDH-BS and GluDH-BA on their activity and stability, we compared these variations with previous mutagenesis studies. Only P45A mutation has been previously reported to enhance activity and stability of GluDH-BS, even though the location of this amino acid is further away from the active site and subunit interface (Vázquez-Figueroa et al. 2007). Also, a single mutation of amino acids outside the active site channel (i.e. F155Y and E170 K) in GluDH-BS has been shown to affect both activity and stability significantly (Vázquez-Figueroa et al., 2007). From these evidences, it is highly likely that the differences in the sequences, even though at amino acids on the surface or remote from the catalytic center, can lead to the activity and thermostability alteration. To further our understanding of the structure–function relationship, mutational analysis of other positions is now in progress.

### Expression and purification of GluDH-BA

**Fig. 4:**
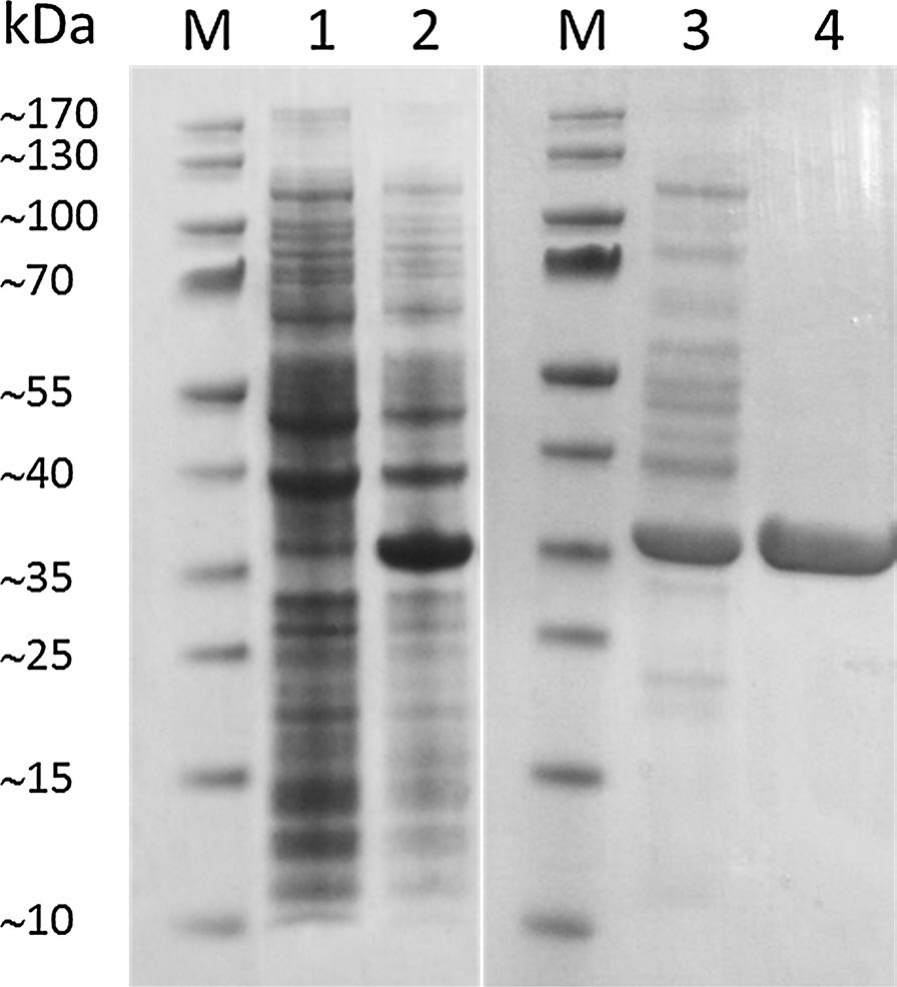
SDS-PAGE showing an expression of GluDH-BA in *E. coli* BL21(DE3). *Lane*; *M*, protein marker (PageRuler™ Prestained Protein Ladder, Thermo Scientific); *1* whole-cell without an induction; *2* whole-cell grown in an autoinduction medium at 37 °C for 6 h and then at 20 °C for 14 h; *3* soluble fraction prepared from *2*; and *4* purified GluDH-BA eluted from Ni-column. *Lane 3* and *4* contained 10 µg of protein per lane

To simplify the enzyme purification, the recombinant GluDH-BA (with 6x His-tag at the N-terminal) was expressed in *E. coli* BL21(DE3). Based on the activity of the purified enzyme (123 U/mg-protein), GluDH-BA contributed to approximately 31 % of total intracellular proteins and the purified GluDH-BA could be obtained from the crude extract (38.4 U/mg-protein) with high yield (35 %) and purity (Fig. 4). The observed size from SDS-PAGE was, however, larger than the expected size of GluDH-BA (28 kDa) possibly as a result of the 6x His-tag as well as the fact that ‘gel shifting’ phenomenon, in which the migration on SDS-PAGE does not correlate with formula molecular weight, was affected by tertiary structure of the migrated protein (Rath et al. 2009). Also, GlcDH-BS (28.1 kDa) was previously reported to appear as a band of approximately 33 kDa by Wang et al. (2013). It should be noted that the productivity of GluDH obtained in this study (8.42 mg-GluDH/g-wet cells; 1035 U/g-wet cells) is among the highest productivity reported for GluDH produced by recombinant *E. coli* (1000–1300 U/g-wet cells; Xu et al. 2007). The purified GluDH-BA was stable for at least 4 months when stored at −80 °C in CPB buffer (pH 6) (data not shown).

### Effect of pH on the activity and stability of GluDH-BA

Despite a widespread use of Tris–Cl buffer (pH 7.2–9.0) and potassium phosphate buffer (pH 5.0–8.0) for GluDH activity assay (Fujita et al. 1977; Boontim et al. 2004; Weckbecker and Hummel 2005), in this research, buffers with a wider pH range such as Britton-Robinson buffer (pH 2.6–11.8) and Citrate–Phosphate-Borate or CPB buffer (pH 2.0–12.0) were tested and compared with a commonly used Tris–Cl in order to eliminate the effect of buffer types in a study on the effect of pH. All three buffers were evaluated at pH 8, an optimum pH generally reported for GluDH activity assay, using GluDH-BA. While Britton-Robinson buffer resulted in a significantly lower GluDH activity (58 % of that observed in Tris–Cl buffer), CPB buffer was found to be comparable to Tris–Cl (not significantly different at α = 0.05) and therefore was suitable for a study on the effect of pH. Moreover, as Tris–Cl was reported to interfere with the Bradford dye used for protein assay (Stoll and Blanchard 1990), CPB was used throughout the rest of this study.

**Fig. 5:**
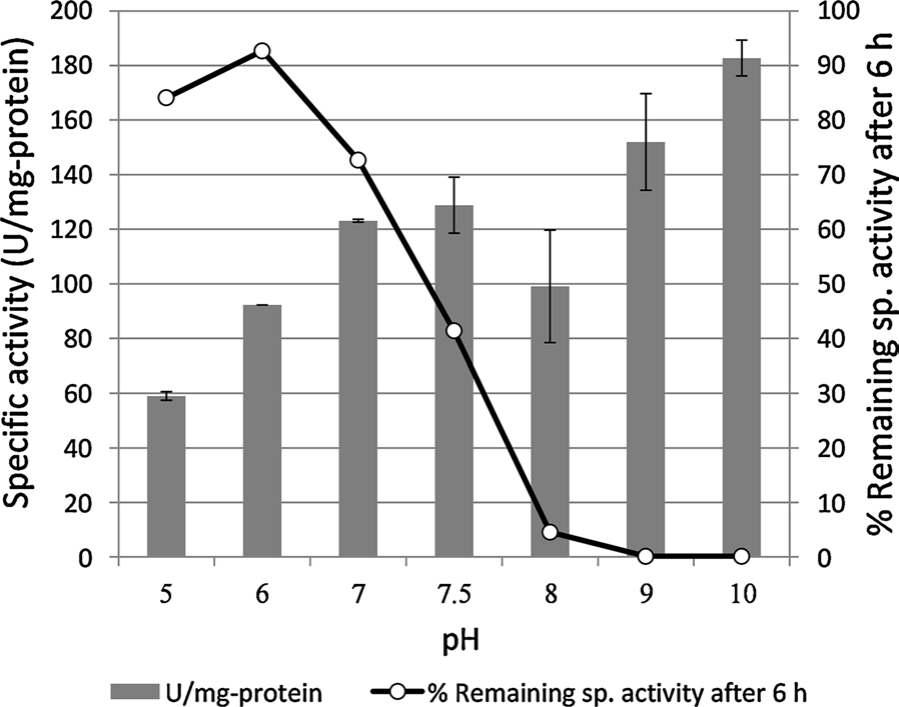
Effect of pH on specific activity and stability of purified GluDH-BA. The activity was assayed at 37 °C using CPB buffer with a specified pH. *Vertical bars* show the standard deviation of the mean based on two independent replicates. Stability is presented as % remaining specific activity after incubating in CBP buffer with a specified pH at 30 °C for 6 h

**Fig. 6:**
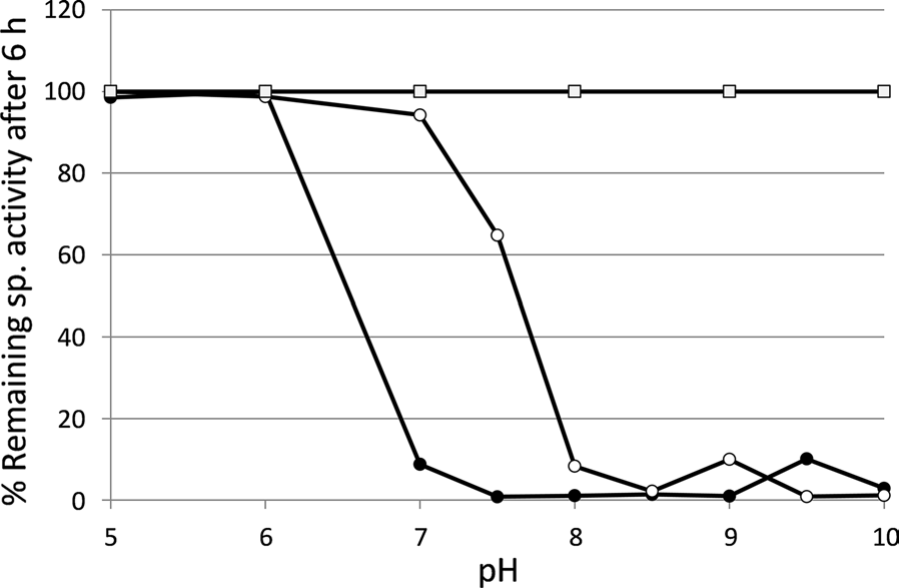
Influence of pH on stability of crude GluDH-BS (*closed circle*) and GluDH-BA (*open circle*) when using NADP^+^ as a cofactor. The activity was determined using CPB with a specified pH at 37 °C after 6 h of incubation at 30 °C. Stability of in vivo GluDH-BA inside the whole-cell *B. subtilis* BA (*open square)* was determined from the cell free extract prepared after the whole-cell biocatalyst was exposed to CPB with a specified pH at 30 °C for 6 h. Relative activity (%) was calculated based on the maximum activity observed at pH 5

The optimum pH for purified GluDH-BA was determined by measuring the activity at 37 °C using CPB buffer over the pH range of 5–10. As a result, the optimal pH of the purified GluDH-BA was observed at pH 10 (Fig. 5), similar to that of an alkali-resistant GluDH from *L. sphaericus* G10 (Ding et al. 2011) but higher than pH 8 generally reported for GluDHs from *B. subtilis* (Fujita et al. 1977; Hilt et al. 1991; Weckbecker and Hummel 2005) and *B. thuringiensis* (Boontim et al. 2004). In this study, effect of pH on stability was evaluated by incubating the enzyme at a specified pH (30 °C for 6 h) prior to an activity assay at 37 °C. Stability is presented as a percentage of remaining specific activity after 6 h (Fig. 6). GluDHs from *Bacillus* species were generally unstable at a pH higher than 8 (Mitamura et al. 1989; Hilt et al. 1991; Nagao et al. 1992; Boontim et al. 2004) as a result of repulsion of the acidic amino acids located at the subunit–subunit interface (Nagao et al. 1989). Similar to other GluDHs from *Bacillus* species, purified GluDH-BA was highly stable in acidic condition (pH 5–6) but was completely inactivated at pH ≥ 8.

It should be noted that, in this study, an unusually long test period of 6 h was employed instead of a commonly used period of 0.5–1 h, mainly because the bioconversion in which the enzyme will be applied are generally conducted for a long period (e.g. 3–24 h). Therefore, the conclusion drawn from this study may not agree well with that reported in a study using a shorter incubation period. For example, while GluDH from *B. subtilis* was previously reported to withstand up to pH 8 with a test period of 20 min (Fujita et al. 1977), the crude GluDH-BS (prepared from a recombinant *B. subtilis* BS) tested in this study was stable only up to pH 6 when the test period of 6 h was used (Fig. 6). This hypothesis was confirmed when the activity of crude GluDH-BS was monitored over time at pH 8 (Table 4). The specific activity observed decreased significantly after 30 min of incubation at pH 8. Crude GluDH-BA (prepared from a recombinant *B. subtilis* BA), on the other hand, could withstand up to pH 7.5 (Fig. 6), similar to the purified GluDH-BA described previously (Fig. 5). Therefore, in order to obtain useful information for practical applications, the test period used for evaluation of enzyme stability should be prolonged (≥1 h).

**Table 4:** % Relative in specific activity of crude GluDH-BS determined after incubation in CPB (pH 8, 30 °C) for a specified period

Time of exposure (h)	% Relative of specific activity^A^
0	100 ± 0^a^
0.5	73.8 ± 14.9^ab^
1	41.2 ± 15.5^b^
3	1.3 ± 0.6^c^
6	0 ± 0^c^

Different superscripts indicate a significant difference between treatments at 95 % confidence level (*p* value ≤0.05)

^A^The GluDH activity was determined at 37 °C

For bioconversion of toxic chemicals, tolerance of the genetically engineered host towards such chemicals was highly critical (Schewe et al. 2008; Siriphongphaew et al. 2012). When compared with *E. coli* DH5α, *B. subtilis*168 exhibited significantly higher tolerance towards toxic chemicals (Siriphongphaew et al. 2012) and therefore was selected for development of a whole-cell cofactor regenerator in this study. To investigate the effect of extracellular pH on the stability of intracellular GluDH-BA, cells of a recombinant *B. subtilis* BA were incubated in CPB with a specified pH for 6 h and the specific activity of their cell-free extracts were then compared with that of a control (a cell-free extract prepared from the cells without an exposure). As expected, the specific GluDH activity remained unchanged regardless of the extracellular pH that the cells were exposed to (Fig. 6).

### Effect of temperature on the activity and stability of GluDH-BA

**Fig. 7:**
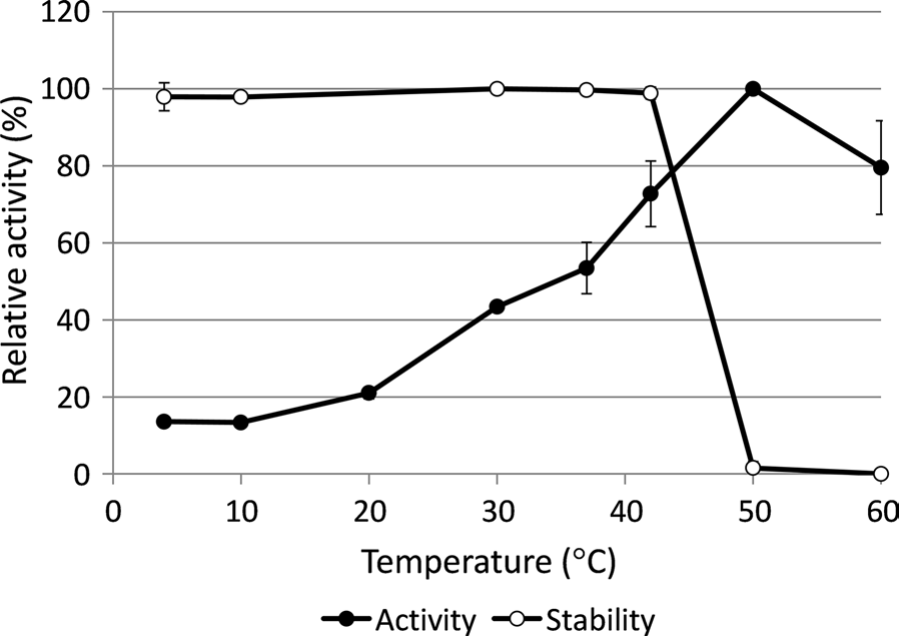
Effect of temperature on relative specific activity and stability of purified GluDH-BA. The activity was determined using CPB (pH 7) with at NADP^+^ as a cofactor 37 °C. For stability, the remaining activity was determined after 6 h of incubation at a specified temperature

Similar to other GluDHs reported for *Bacillus* species, purified GluDH-BA exhibited maximum activity at 50 °C but was found to be unstable at this temperature (Fig. 7). Surprisingly, a relatively high specific activity (80 %) could still be observed at 60 °C where most *Bacillus* GluDHs exhibited almost no activity (Yamamoto et al. 1990; Nagao et al. 1992; Boontim et al. 2004).

### Effect of organic solvents on the stability of GluDH-BS and GluDH-BA

According to the obtained result, purified GluDH-BA exhibited higher stability towards organic solvents than GluDH-BS, especially in the case of 1- and 2-hexanol (Table 5). Both GluDHs could tolerate high concentration of *n*-hexane, despite the fact that 50 % (v/v) *n*-hexane has been reported to completely eliminate the activity of an organic solvent-tolerant GluDH-Ls from *Lysinibacillus sphaericus* G10 after a 1-h exposure (Ding et al. 2011). The fact that GluDH-BA could withstand a high concentration of *n*-hexane and its respective alcohols makes GluDH-BA a good candidate for cofactor regeneration in a hydroxylation of *n*-hexane.

**Table 5:** Stability of purified GlcDH-BS and GlcDH-BA in various organic solvents

Solvents	logPo/w	% Relative activity^a^			
		GluDH-BS		GluDH-BA	
		10 % v/v	50 % v/v	10 % v/v	50 % v/v
DMSO	−1.4	87 ± 3	98 ± 3	100 ± 0	99 ± 1
Acetone	−0.2	80 ± 2	1 ± 1	99 ± 1	1 ± 1
Ethanol	−0.2	86 ± 3	1 ± 1	100 ± 1	19 ± 16
*n*-Butanol	0.9	0 ± 0.5	0 ± 0	2 ± 0	1 ± 1
*n*-Hexane	3.8	89 ± 0	99 ± 1	100 ± 0	100 ± 0
1-Hexanol	1.8	56 ± 10	n.a.	94 ± 0	n.a.
2-Hexanol	1.8	68 ± 4	69 ± 1	95 ± 1	93 ± 2

The relative activity was calculated based on an activity of a control without organic solvent (water was added instead)

*n.a.* data not available

^a^The activity was determined using CPB (pH 6) at 37 °C after an exposure with a specified organic solvent at 30 °C for 1 h

### Kinetic constants

Unlike GluDH-BS which equally preferred NAD^+^ and NADP^+^ as a cofactor, a purified GluDH-BA exhibited higher preference towards NADP^+^ (Table 3), similar to GluDHI, GluDHII and GluDHIWG3 from *B. megaterium* (Mitamura et al. 1989; Yamamoto et al. 1990). In comparison with GluDH-BS (42.9 mM), a significantly lower *K*_M_-value towards glucose (5.5 mM) was observed for GluDH-BA, suggesting its ability to immediately react with a small amount of glucose present in vivo. Faster NAD(P)H regeneration can be achieved at lower glucose concentration for the same amount of GluDH. Therefore, a high expression level may not be required, making GluDH-BA suitable for enzymatic cascading. Moreover, for an in vivo application in a whole-cell biocatalyst, an enzyme with low *K*_M_ for NAD(P)^+^ would be most appropriate due to the low NAD(P)^+^ concentration within the cell. Nonetheless, the reaction rate would depend on the turnover number of the enzyme. For this reason, GluDH from *Sulfolobus solfataricus*, despite having a low *K*_M_ for NAD(P)^+^, is probably not suitable for in vivo applications due to its low turnover number (Table 3).

For applications in a form of crude lysate and purified enzyme, an enzyme with high *K*_M_ (possibly for glucose but not necessary for NAD^+^) and high k_cat_ would be most preferable. Such kinetic properties would allow for an operation under high substrate concentration (glucose) and therefore fast reaction rate (dictated by the k_cat_) could be obtained. GluDH with low *K*_M_ towards glucose, including GluDH from *S. solfataricus,* is more likely to suffer from glucose inhibition than other GluDHs. Nonetheless, for industrial and synthesis purposes, kinetic parameters alone should not be used to identify which enzyme is suitable for catalysis. Stabilities and inhibitions should also be considered. Since GluDHs are widely used as cofactor regeneration enzyme, it would be useful to have a wide range of GluDHs to select from. This is because certain variants may be inhibited or incapable in a certain NAD(P)H-consuming reaction.

### Application as a whole-cell cofactor regenerator for hydroxylation of *n*-hexane

In a recombinant *B. subtilis* BA, GluDH-BA was expressed at a high level (6.24 U/mg-total protein) comparable to the GluDH activity obtained in *E. coli* when using the pET system (7.18 U/mg-total protein; Richter et al. 2010) or that expressed in *B. subtilis* under a constitutive strong promoter P43 (4.9 U/mg-total protein; Zhu et al. 2006). In this study, *B. subtilis* BA was evaluated as a whole-cell cofactor regenerator in a coupling whole-cell system for hydroxylation of *n*-hexane.

**Fig. 8:**
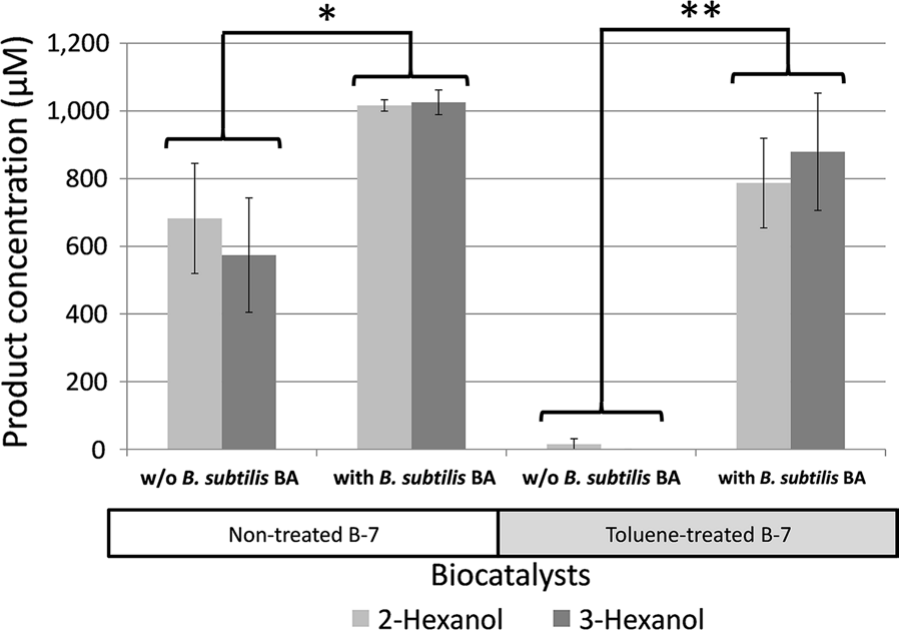
Products of hexane bioconversion, 2-hexanol (*light grey bar*) and 3-hexanol (*dark grey bar*), obtained using toluene-treated or non-treated whole-cell biocatalysts with or without an incorporation of *B. subtilis* BA. Bioconversion was performed with 100 mM hexane using resting cells (OD_600_ = 25 for each biocatalyst) suspended in 50 mM phosphate buffer (pH 7) supplemented with 110 mM glucose at 30 °C for 3 h. For toluene-treated biocatalyst, 0.2 mM NADP^+^ was supplemented into the reaction. Vertical bars show the standard deviation of the mean based on two independent replicates. * and ** indicate a statistically significant difference at α = 0.1 and α = 0.05, respectively

Coupling whole-cell system, first demonstrated by Zhang et al. (2009), uses two types of permeabilized biocatalysts expressing different enzymes, one for production of desired product and another for regeneration of required cofactor. Each reaction occurs in a separate host cell while the cofactors migrate between the two permeabilized cells. Although *n*-hexane is a small molecule that does not require cell permeabilization, it was chosen as our model substrate so that we can also evaluate the system with non-permeabilized biocatalyst as well. When coupling with a recombinant *B. subtilis* 3C5N (pHP2NB) expressing P450 BM3 F87V (referred as *B. subtilis* B-7), *B. subtilis* BA enhanced the production of 2- and 3-hexanol significantly (Fig. 8). The fact that *B. subtilis* BA enhanced the bioconversion even when both biocatalysts were not permeabilized suggested that glucose and NADP(H) could already gain access into the cells. Nonetheless, a small amount of hexane used as a reaction substrate might play a part in membrane permeabilization and therefore could not be ruled out.

Permeabilization by a toluene treatment (at 1 % v/v) was frequently employed in bioconversion to guarantee access of substrate and cofactor into the cells (Cánovas et al. 2005; Zhang et al. 2009). Unfortunately, toluene treatment resulted in an absolute loss of indigenous cofactors inside the cells and extracellular supplementation of NAD(P)^+^ was necessary. Permeabilized *B. subtilis* B-7 failed to catalyze the hexane hydroxylation even when 0.2 mM NADP^+^ was supplemented extracellularly, agreeing with an extremely low GluDH activity observed previously (Table 2). Interestingly, an incorporation of a non-permeabilized whole-cell *B. subtilis* BA could effectively restore the bioconversion (Fig. 8).

## Discussions

In this study, NAD(P)-dependent GluDH from *B. amyloliquefaciens* SB5 (GluDH-BA) has been successfully cloned and expressed in both *E. coli* and *B. subtilis*. Several strains of *B. amyloliquefaciens* have been widely regarded as a member of ‘plant growth promoting rhizobacteria’ or PGPR (Idriss et al. 2007). The mechanisms by which PGPR can exert a positive effect on plant growth include the solubilization of insoluble inorganic phosphate compounds (such as tricalcium phosphate and hydroxyapatite) via an action of organic acids synthesized by them. Considering the fact that gluconic acid (formed via an action of glucose dehydrogenase) was reported to be the most common agent for mineral phosphate solubilization, it was not surprising that *B. amyloliquefaciens* with a high level of GluDH activity has been identified as one of the most powerful inorganic phosphate solubilizers (Rodriguez and Fraga 1999). High level of GluDH activity observed in *B. amyloliquefaciens* SB5 (Table 2) agreed well with those findings and the phosphate solubilizing property of *B. amyloliquefaciens* SB5 is now being investigated in detail.

Comparing to GluDHs reported from genus other than *Bacillus*, the specific activity of the purified GluDH-BA (123 U/mg-protein) was comparable to that reported for *G. suboxydans* (Adachi et al. 1980), but lower than that of *L. sphaericus* G10 (Ding et al. 2011) and *S. solfataricus* (Giardina et al. 1986) (Table 3). Nonetheless, it should be noted that a specific activity value highly depends on several factors (i.e. buffer, pH and substrate concentration) and therefore may not be a good parameter for comparison. Despite a highly similar sequence with GluDH-BS, GluDH-BA exhibited significantly higher specific activity (4.7-fold) and stability when pH was higher than 6. GluDH-BA also exhibited higher tolerance towards organic solvents, especially hexanols.

Amino acid sequence alignment and structural analysis of both GluDHs revealed several novel potential amino acid positions [e.g. position 16 in the region of NAD(P)^+^-binding motif and position 95 in the active site channel] for further improvement of the enzyme activity and stability via site-directed mutagenesis. The 3D structure of GluDH IV from *B. megaterium* (Nishioka et al. 2012) revealed that amino acid position 16 was a part of the outer layer of the active site channel located at least 16 Å away from the reactive center. The difference in size and chemical properties of an amino acid at this position may account for the difference in enzyme activity and stability. Despite the fact that a single mutation of amino acids outside the active site channel in GluDH-BS affected both activity and stability significantly (Vázquez-Figueroa et al. 2007), similar phenomenon can be observed as a result of accumulated mutations at scattered locations as well (e.g. an enzyme-surface mutant Q42E, P45A and N46A in *B. subtilis*; Vázquez-Figueroa et al. 2008).

High specific activity as well as low *K*_M_-value towards glucose and NADP^+^ of GluDH-BA suggested its potential for in vivo applications. While the high specific activity allows for an efficient cofactor recycling even with a low level of expression (exerting lower metabolic burden onto the cells and making it more suitable for enzymatic cascading), the low *K*_M_-value, on the other hand, allows the enzyme to immediately react with a small amount of glucose and NADP^+^ present in vivo. As GluDH-BA was successfully co-expressed along with the enzyme P450 BM3 in our previous study (Siriphongphaew et al. 2012), in this study, a whole-cell *B. subtilis* overexpressing GluDH-BA alone was evaluated as a cofactor regenerator in a coupling whole-cell system instead. Coupling whole-cell system offers several advantages including process flexibility as well as an opportunity to supplement additional cofactor externally. The enzymes-of-interest can be expressed in their most appropriate hosts and the enzyme activity level required for optimal bioconversion can be adjusted easily by adjusting the amount of each whole-cell biocatalyst used. Moreover, as a whole-cell biocatalyst generally maintains its cytoplasmic pH near neutrality via several acid and alkaline pH homeostasis (Booth 1985; Padan et al. 2005; Baker-Austin and Dopson 2007), the intracellular enzyme (GluDH-BA in our case) is well protected from an extreme pH outside the cells. This allows for longer use period of the biocatalyst as well as higher process flexibility (i.e. wider range of possible reactions, ease of process control). Although *B. subtilis* BA has been proven as an effective cofactor regenerator for a coupling whole-cell system in this study, for practical applications, it should be investigated further on its ability to be recycled or stored as a frozen cell pellet.
